# Perceived changes in health behaviours and body weight in response to a cancer diagnosis among individuals living with and beyond breast, prostate, and colorectal cancer in the UK: a cross-sectional study

**DOI:** 10.1007/s00520-025-09305-z

**Published:** 2025-03-04

**Authors:** Francisco Brenes-Castillo, William Goodman, Phillippa Lally, Abi Fisher, Rebecca J. Beeken

**Affiliations:** 1 Research Department of Behavioural Science and Health, University College London, Gower Street, London, WC1E 6BT UK; 2 Institute of Psychiatry, Psychology & Neuroscience, King’s College London, London, SE5 8AB UK; 3 Leeds Institute of Health Sciences, School of Medicine, University of Leeds, Leeds, LS2 9JT UK; 4 School of Psychology, University of Surrey, Guildford, Surrey, GU2 7XH UK

**Keywords:** Alcohol, Body weight, Cancer diagnosis, Diet, Perceptions, Physical activity

## Abstract

**Purpose:**

This study explored perceived changes in health behaviours and body weight following a cancer diagnosis and investigated related sociodemographic and clinical characteristics.

**Methods:**

Individuals living with and beyond breast, prostate, or colorectal cancer (*N* = 5835) completed the ‘Health and Lifestyle After Cancer Survey’ which explored their perceptions of health behaviour change following a cancer diagnosis. Multinomial logistic regressions were conducted with perceived changes as dependent variables, and sociodemographic and clinical characteristics as independent variables.

**Results:**

Approximately half of the participants did not perceive changes in their physical activity, alcohol intake and body weight, and most did not perceive dietary changes. Less than a third of individuals perceived positive health behaviour changes (11.7% increased physical activity, 24.3% healthier diet, and 31.3% decreased alcohol intake), 35.9% perceived decreases in physical activity, and 27.0% perceived increases in body weight, whereas 19.2% perceived decreases in body weight. Individuals with no education, who were unmarried, and with anxiety/depression and pain/discomfort, were more likely to perceive changes in physical activity, body weight, and diet, but in different directions. Participants of younger age were more likely to perceive increases in their physical activity, a healthier diet, and increases in body weight.

**Conclusion:**

Following a diagnosis of cancer, a large proportion of individuals perceived that their health behaviours were unchanged. However, some groups of individuals were more likely to perceive positive changes, whereas others were more likely to perceive negative changes, with differences also observed according to the type of health behaviour.

Implications for cancer survivors.

Participants with no education, who were unmarried, with anxiety/depression and pain/discomfort, may be more at risk of experiencing negative health behaviour changes post-diagnosis. Clinicians should consider targeting health behaviour support to prevent worse outcomes in the long term.

**Supplementary Information:**

The online version contains supplementary material available at 10.1007/s00520-025-09305-z.

## Introduction

Globally, 19.3 million cancer cases were diagnosed in 2020, with breast, colorectal, and prostate cancer being among the most prevalent [1]. Earlier diagnoses and more efficient treatments have led to steadily declining cancer mortality rates [2]. In England and Wales, 50% of individuals diagnosed with cancer will live for 10 years or more [3]. However, individuals living with and beyond cancer (LWBC) may experience long-term sequelae such as other chronic health conditions, pain, cognitive impairment, depression, and anxiety [4]. Research has shown that adhering to positive health behaviours can help minimise these long-term consequences and improve overall quality of life [5, 6].

The World Cancer Research Fund (WCRF) recommends that individuals LWBC adhere to the same health behaviour guidelines as those for cancer prevention. These guidelines state that individuals should maintain a healthy body weight (Body Mass Index > 18 kg/m^2^ and < 25 kg/m^2^), be physically active (at least 150 min of moderate to vigorous physical activity per week), limit alcohol consumption, and have a healthy diet (e.g., by consuming less red meat and processed foods, and consuming more vegetables and fruits) [7].

Longitudinal studies have generally found negative or no health behaviour changes following a cancer diagnosis. A secondary data analysis study from the English Longitudinal Study of Ageing compared health behaviours between participants who were diagnosed with cancer (*n* = 433) and participants without cancer (*n* = 4713), and concluded that participants diagnosed with cancer were less physically active, more sedentary, and had the same alcohol intake patterns when compared to participants without cancer [8]. Furthermore, a 6-year longitudinal study from Denmark, concluded that 449 breast cancer survivors had no differences in their body weight or alcohol intake patterns following a cancer diagnosis [9]. Similarly, a 12-year longitudinal study from Canada among 497 individuals LWBC found no difference in alcohol intake, diet, or physical activity following a cancer diagnosis [10]. A longitudinal study from Australia followed 153 breast cancer survivors for 15 years and found no long-term positive changes in their health behaviours [11]. On the other hand, a study from Portugal prospectively followed 428 breast cancer patients for 3 years and found some positive health behaviour changes after their cancer diagnosis, e.g., 24.6% reduced their alcohol consumption and 9.9% became physically active [12].

Cross-sectional and qualitative studies also suggest that a cancer diagnosis may be perceived as contributing to positive health behaviour changes [13–15]. A cross-sectional study interviewed 356 individuals recently diagnosed with cancer and found that 40.4% reported improving their diet and 20.8% reported increasing their physical activity [16]. Similarly, a qualitative study with 19 cancer survivors in the UK found that participants reported improving their diet post diagnosis to improve their general health [17]. A cross-sectional study from the United States with 315 breast cancer survivors found that the majority (84%) reported a positive change for at least 1 nutrition or physical activity behaviour post-diagnosis, while also reporting a number of diagnosis and treatment-related barriers to engaging in these behaviours [18].

Perceptions of change may not be a completely accurate reflection of actual behaviour [19], but could indicate someone's readiness to receive and/or act on health behaviour advice. Those who perceive negative changes may be more receptive to interventions, whereas individuals perceiving positive changes may be less interested in support [20]. Perceptions of change may also reflect readiness towards actual behaviour change and the perceived consequences of changing the behavior. Individuals typically perceive greater impact from increasing behaviours compared to decreasing those behaviours [21]. Perceptions may also be an important indicator of someone’s overall well-being and health. For example, studies exploring perceptions of ageing suggest that individuals who perceive negative changes with ageing are also more likely to have worse health behaviours and be in poorer health [22]. Understanding how different patients perceive their behaviour to have changed, could inform how health professionals approach health promotion conversations in their clinical practice, and help to identify individuals at risk of poorer outcomes.

Certain demographic and clinical characteristics may contribute to differences in perceptions of change. For example, a secondary data analysis with the ASCOT database found that younger age, female gender, non-white ethnicity, not being married/cohabiting, and having two or more comorbidities was related to increased odds of perceptions of needing to make dietary changes [23]. However, there is a paucity of information relating demographic and clinical characteristics to perceived changes in health behaviours and body weight following a cancer diagnosis.

To our knowledge, no previous study has examined perceived changes in health behaviours and body weight following a cancer diagnosis within a large sample of individuals LWBC in England. The aim of this study was to assess perceived changes in physical activity, diet, alcohol intake, and body weight among individuals LWBC following a cancer diagnosis. This study explored potential sociodemographic and clinical characteristics that were associated with the perceived changes, since these could be used to target future behaviour change interventions among individuals LWBC.

## Methods

### Design

This study consisted of a secondary data analysis on the Health and Lifestyle after Cancer Survey, a comprehensive cross-sectional survey of beliefs and behaviours, conducted among individuals LWBC [24].

### Procedure and ethics

Participants were recruited between 2015 and 2018 from ten NHS (National Health Service) Trusts in London and Essex (United Kingdom). Participants were identified by hospital staff and then sent a paper version of the survey and a link to an online version, which they could return to the research centre in a freepost envelope, or complete online. Ethical approval for the Health and Lifestyle after Cancer Survey was provided through the Oxford B Research Ethics Committee (reference number 14/SC/1369). Participants were informed that survey completion indicated informed consent [24].

### Participants

Participants were eligible for the current study if they were > 18 years old and had been diagnosed with breast, colorectal, or prostate cancer. The inclusion criteria were deliberately broad to reduce burden at hospital sites, and to gather as wide a range of views as possible [24].

### Measures

All measures were collected via self-report survey, and the following were included:

#### Primary outcome variable: perceived changes

Questions about perceived changes were created specifically for the survey by the study authors. Three questions with 3 items each asked participants to consider whether their physical activity, alcohol intake, and body weight had increased, decreased, or stayed the same following their cancer diagnosis.

The perceived change in diet variable asked participants if they thought their diet was healthier, unhealthier or stayed the same following a cancer diagnosis (3 items).

#### Sociodemographic and clinical characteristics

Age, sex (female and male), marital status (unmarried (a combination of separated, widowed, single and divorced) vs married/cohabiting), ethnicity (collected using 16 subcategories but for this analysis, dichotomised into white or non-white due to small numbers in some groups), current employment (collected using 8 subcategories but dichotomized into yes (employed) vs. no (not employed)), and educational level (none vs. GCSE to A-levels or equivalent vs. undergraduate degree level or above).

The following cancer variables were asked considering their most recent cancer. Type of cancer (breast, prostate, or colorectal). Number of comorbidities was calculated from participants ticking if they had a number of conditions from a pre-generated list (15 options) and also counting the number of conditions they wrote in a free text box if they ticked that they had additional conditions; this was then categorised into none, one, two or three or more. Cancer spread (yes vs no), and number of cancer treatments (none vs. one vs. two vs. three and above) were assessed. Anxiety/depression was assessed on a 5-item scale (“1 = not anxious or depressed” to “5 = extremely anxious or depressed) and dichotomised into yes (slightly or moderately or severely or extremely anxious or depressed) vs. no (not anxious or depressed). Pain/discomfort (“1 = no pain or discomfort” to “5 = extreme pain or discomfort) was also dichotomised into yes (slight or moderate or severe or extreme pain or discomfort) vs. no (no pain or discomfort). Both questions were taken from the EQ5D questionnaire[25].

### Statistical analysis

Statistical analysis was conducted through the IBM SPSS Statistics software [26]. Descriptive statistics determined the prevalence of participants who perceived that their health behaviours, physical activity, alcohol consumption, or body weight increased, decreased, or stayed the same, and the diet had become healthier, unhealthier, or stayed the same, following a cancer diagnosis.

Missing data was assessed to determine if imputation was required. Little’s MCAR test *X*^*2*^ (0) = 65.5, *p* = < 0.001, indicated that the data between those with complete and missing data may have not been missing at random. Missing data for outcomes and characteristics was addressed using multiple imputation to reduce bias [27, 28]. Sociodemographic and clinical characteristics were included as predictors with and without the missing data and were included in the imputation. Ten iterations were run.

Multinomial logistic regressions were conducted to explore which sociodemographic (age, sex, marital status, ethnicity, employment, and education) and clinical (number of comorbidities, anxiety/depression, pain/discomfort, cancer spread, and number of cancer treatments) characteristics were associated with perceived changes in health behaviours (physical activity, diet, alcohol) and in body weight following a cancer diagnosis. Type of cancer was not included in the analyses due to the potential for multicollinearity since it is identical to gender in the breast cancer (all female) and prostate cancer (all male) subcategories [29].

Sensitivity analyses were run in the imputed data set and among those with complete data and were compared to assess the similarity of responses.

## Results

### Sample characteristics

The survey was mailed to 13,500 individuals LWBC and returned by 5835 individuals (43% response rate). Of 5835 responses, 5801 (99.4%) were completed in paper format and 34 (0.6%) were completed online. Participant characteristics are shown in Table 1. Participants had a mean age of 67.4 years (*SD* = 11.8), were mostly female (56.0%), white (90.0%), married/cohabiting (69.2%), achieved GCSE or vocational qualifications (61.2%), and were not working (70.2%). Regarding clinical characteristics, most participants reported that their cancer had not spread (77.1%), had none or one comorbidity (65.8%), reported feeling pain/discomfort (59.5%), and reported not being anxious/depressed (55.7%).

**Table 1:** Descriptive statistics for sociodemographic and clinical characteristics in individuals LWBC (*N* = 5835)

Characteristics	***N (%)***
Sociodemographic characteristics	
Age, Mean (SD)	67.4 (11.8)
Missing	36 (0.01)
Sex	
Male	2553 (43.8)
Female	3266 (56.0)
Missing	16 (0.3)
Ethnicity	
White	5249 (90.0)
Any other ethnicity	554 (9.5)
Missing	32 (0.5)
Highest level of education	
None	1709 (29.3)
GCSE/vocational	1613 (27.6)
A level	584 (10.0)
Degree	1379 (23.6)
Missing	550 (9.4)
Marital status	
Married/cohabiting	4037 (69.2)
Unmarried	1781 (30.5)
Missing	17 (0.3)
Employment	
Unemployed	4097 (70.2)
Employed	1684 (28.9)
Missing	54 (0.9)
Clinical characteristics	***N (%)***
Cancer type	
Breast	2786 (47.7)
Prostate	1839 (31.5)
Colorectal	1210 (20.7)
Missing	0
Cancer spread	
Yes	558 (9.6)
No	4498 (77.1)
Missing/don’t know	779 (13.4)
Number of comorbid conditions	
0	1849 (31.7)
1	1991 (34.1)
2	1120 (19.2)
3 or more	875 (15.0)
Missing	0 (0)
Number of treatments	
0	292 (5.0)
1	1857 (31.8)
2	1881 (32.2)
3 or more	1696 (29.1)
Missing	109 (1.9)
Pain/Discomfort	
Yes	3472 (59.5)
No	2139 (36.7)
Missing	224 (3.8)
Anxiety/Depression	
Yes	2364 (40.5)
No	3249 (55.7)
Missing	222 (3.8)

*N* number of participants, *SD* standard deviation

Table 2 shows descriptive statistics for the perceived changes in physical activity, diet, alcohol intake, and body weight. Less than a third of individuals perceived positive health behaviour changes (11.7% increased physical activity, 24.3% healthier diet, and 31.3% decreased alcohol intake), others perceived negative health behaviour changes (4.1% unhealthier diet, 2.8% increased alcohol intake, and 35.9% decreased physical activity), and others perceived changing body weight (27.0% perceived increases and 19.2% perceived decreases). Approximately half of individuals did not perceive changes in their physical activity (50.6%), alcohol intake (52.5%) and body weight (52.6%), whereas 70.8% did not perceive changes in their diet.

**Table 2:** Descriptive statistics for perceived changes following a cancer diagnosis in individuals LWBC (*N* = 5835)

Perceived changes following a cancer diagnosis	*N (%)*
Physical activity	
Increased	681 (11.7)
No change	2954 (50.6)
Decreased	2095 (35.9)
Missing	105 (1.8)
Diet	
Healthier	1416 (24.3)
No change	4131 (70.8)
Unhealthier	239 (4.1)
Missing	49 (0.8)
Alcohol intake	
Increased	166 (2.8)
No change	3061 (52.5)
Decreased	1825 (31.3)
Missing	783 (13.4)
Body Weight	
Increased	1578 (27.0)
No change	3068 (52.6)
Decreased	1123 (19.2)
Missing	66 (1.1)

*N* number of participants

### Sociodemographic characteristics related to perceived changes

Individuals who were unmarried (OR = 1.31, 95% CI = 1.14–1.49) and with no education (OR = 1.41, 95% CI = 1.18–1.68), had increased odds of perceiving decreased physical activity. Those with no education (OR = 0.65, 95% CI = 0.50–0.84) were less likely to perceive increased physical activity and those who were unemployed (OR = 1.51, 95% CI = 1.21–1.88) had higher odds of perceiving increased physical activity. Individuals who were white (OR = 0.49, 95% CI = 0.40–0.60) had decreased odds of perceiving a healthier diet, and unmarried participants (OR = 2.20, 95% CI = 2.20, CI = 1.66–2.90) had increased odds of perceiving an unhealthier diet (Table 3).

Being a male (OR = 1.44, 95% CI = 1.25–1.67; OR = 1.30, 95% CI = 1.11–1.51) with no education (OR = 1.63, CI = 1.34–1.97; OR = 1.31, 95% CI = 1.09–1.58) was related to higher odds of perceiving reduced alcohol intake and with higher odds of perceiving increased body weight respectively. Having white ethnicity (OR = 0.50, 95% CI = 0.41–0.62) was related to decreased odds of perceiving reduced alcohol intake. Individuals with younger age had increased odds of perceiving increases in their physical activity (OR = 0.95, 95% CI = 0.94–0.96), having a healthier diet (OR = 0.96, 95% CI = 0.95–0.97), increasing body weight (OR = 0.96, 95% CI = 0.98–1.01), and increasing alcohol intake (OR = 0.96, 95% CI = 0.94–0.98) (Tables 3–4).

### Clinical characteristics related to perceived changes

Individuals who reported that their cancer had spread (OR = 2.10, 95% CI = 1.68–2.62) and experienced pain/discomfort (OR = 1.85, 95% CI = 1.61–2.13), had higher odds of perceiving decreased physical activity. Those who reported anxiety/depression had increased odds of perceiving both decreased (OR = 1.80, 95% CI = 1.59–2.04) and increased (OR = 1.34, 95% CI = 1.12–1.61) physical activity respectively. Those who reported no comorbidities when compared to three or more (OR = 0.55, 95% CI = 0.45–0.68) and no treatments when compared to three or more (OR = 0.29, 95% CI = 0.20–0.41) had decreased odds of perceiving decreased physical activity. Individuals who reported that their cancer had spread (OR = 2.93, 95% CI = 2.03–4.23), and with pain/discomfort (OR = 2.20, 95% CI = 1.53–3.16) and anxiety/depression (OR = 1.98, 95% CI = 1.49–2.64), had increased odds of perceiving an unhealthier diet. Cancer spread (OR = 1.43, 95% CI = 1.16–1.75) was also related to increased odds of perceiving a healthier diet (Table 3).

**Table 3:** Perceived changes in physical activity and diet following a cancer diagnosis (*N* = 5835)

Characteristics	Perceived increases in physical activity following a cancer diagnosis (no changes as reference)		Perceived decreases in physical activity following a cancer diagnosis (no changes as reference)	
	OR	CI	OR	CI
Sociodemographic (bold as reference)				
Age	**0.95**	**0.94–0.96**	0.99	0.99–1.00
Sex: Female				
Male	0.82	0.66–1.02	1.19	1.03–1.37
Ethnicity: Any other ethnicity				
White	0.77	0.59–1.01	0.83	0.67–1.02
Highest level of education: Degree				
None	**0.65**	**0.50–0.84**	**1.41**	**1.18–1.68**
GCSE/vocational	**0.64**	**0.52–0.80**	1.19	1.00–1.42
A level	0.71	0.52–0.95	1.24	0.99–1.55
Marital status: Married/cohabiting				
Unmarried	1.05	0.87–1.28	**1.31**	**1.14–1.49**
Employment: Employed				
Unemployed	**1.51**	**1.21–1.88**	1.24	1.06–1.46
Clinical (bold as reference)				
Cancer spread: No				
Yes	1.27	0.93–1.75	**2.10**	**1.68–2.62**
Number of comorbid conditions: 3 or more				
0	0.95	0.69–1.30	**0.55**	**0.45–0.68**
1	0.95	0.70–1.30	**0.60**	**0.50–0.73**
2	1.06	0.78–1.47	**0.70**	**0.58–0.85**
Number of treatments: 3 or more				
0	0.78	0.49–1.26	**0.29**	**0.20–0.41**
1	0.97	0.75–1.26	**0.66**	**0.55–0.79**
2	0.92	0.74–1.16	0.82	0.69–0.96
Pain/Discomfort: No				
Yes	0.98	0.83–1.20	**1.85**	**1.61–2.13**
Anxiety/Depression: No				
Yes	**1.34**	**1.12–1.61**	**1.80**	**1.59–2.04**
Characteristics	Perceived healthier diet following a cancer diagnosis (no changes as reference)		Perceived unhealthier diet following a cancer diagnosis (no changes as reference)	
	**OR**	**CI**	**OR**	**CI**
Sociodemographic (bold as reference)				
Age	**0.96**	**0.95–0.97**	0.99	0.98–1.01
Sex: Female				
Male	1.23	1.05–1.43	0.85	0.62–1.17
Ethnicity: Any other ethnicity				
White	**0.49**	**0.40–0.60**	0.58	0.38–0.90
Highest level of education: Degree				
None	0.95	0.78–1.16	1.79	1.17–2.74
GCSE/vocational	0.96	0.81–1.15	1.29	0.84–1.98
A level	0.83	0.67–1.04	1.46	0.88–2.42
Marital status: Married/cohabiting				
Unmarried	1.15	1.00–1.32	**2.20**	**1.66–2.90**
Employment: Employed				
Unemployed	1.07	0.90–1.26	0.99	0.68–1.43
Clinical (bold as reference)				
Cancer spread: No				
Yes	**1.43**	**1.16–1.75**	**2.93**	**2.03–4.23**
Number of comorbid conditions: 3 or more				
0	0.74	0.60–0.91	0.82	0.54–1.24
1	0.76	0.61–0.92	0.64	0.43–0.94
2	0.81	0.65–1.00	0.81	0.54–1.20
Number of treatments: 3 or more				
0	0.61	0.42–0.87	0.52	0.20–1.37
1	0.84	0.70–1.02	1.05	0.70–1.59
2	0.94	0.80–1.11	1.00	0.70–1.44
Pain/Discomfort: No				
Yes	0.99	0.86–1.14	**2.20**	**1.53–3.16**
Anxiety/Depression: No				
Yes	1.12	0.98–1.28	**1.98**	**1.49–2.64**

*OR* odds ratios, *CI* confidence interval. Bold indicates significance at p<0.05 level

Having no treatments (OR = 0.42, 95% CI = 0.30–0.59) when compared to having three or more was related to decreased odds of perceiving reduced alcohol intake. Reporting anxiety/depression was related to higher odds of perceived increases (OR = 2.47, 95% CI = 1.74–3.49) and decreases (OR = 1.34, 95% CI = 1.18–1.52) in alcohol intake. Cancer spread (OR = 1.69, 95% CI = 1.40–2.05) was related to increased odds of perceiving decreases in alcohol intake (Table 4). Reporting cancer spread (OR = 1.49, 95% CI = 1.19–1.85), pain/discomfort (OR = 1.44, 95% CI = 1.25–1.67), and anxiety/depression (OR = 1.36, 95% CI = 1.19–1.56) was related to higher odds of perceiving increases in body weight. Cancer spread (OR = 2.07, 95% CI = 1.65–2.60) was associated with increased odds of perceiving decreases in body weight. Similarly, reporting no comorbidities when compared to three or more (OR = 0.73, 95% CI = 0.59–0.91; OR = 0.45, 95% CI = 0.35–0.56) and no treatments when compared to three or more (OR = 0.25, 95% CI = 0.16–0.37; OR = 0.50, 95% CI = 0.34–0.74) were related to decreased odds in perceiving both increases and decreases in body weight respectively (Table 4). Sensitivity analyses were conducted with the completers dataset and similar results were found (Supplementary Tables 1–2).

**Table 4:** Perceived changes in alcohol intake and body weight following a cancer diagnosis (*N* = 5835)

Characteristics	Perceived increases in alcohol intake following a cancer diagnosis (no changes as reference)		Perceived decreases in alcohol intake following a cancer diagnosis (no changes as reference)	
	OR	CI	OR	CI
Sociodemographic (bold as reference)				
Age	**0.96**	**0.94–0.98**	0.99	0.98–1.01
Sex: Female				
Male	0.90	0.59–1.40	**1.44**	**1.25–1.67**
Ethnicity: Any other ethnicity				
White	0.83	0.39–1.79	**0.50**	**0.41–0.62**
Highest level of education: Degree				
None	0.94	0.54–1.64	**1.63**	**1.34–1.97**
GCSE/vocational	1.03	0.67–1.57	1.06	0.90–1.25
A level	1.09	0.62–1.94	1.11	0.90–1.38
Marital status: Married/cohabiting				
Unmarried	1.47	1.05–2.07	1.09	0.95–1.25
Employment: Employed				
Unemployed	1.32	0.88–1.98	1.05	0.90–1.23
Clinical (bold as reference)				
Cancer spread: No				
Yes	1.45	0.85–2.48	**1.69**	**1.40–2.05**
Number of comorbid conditions: 3 or more				
0	1.50	0.83–2.71	0.91	0.75–1.11
1	1.45	0.84–2.49	0.97	0.80–1.17
2	1.25	0.66–2.34	1.01	0.82–1.25
Number of treatments: 3 or more				
0	1.05	0.40–2.73	**0.42**	**0.30–0.59**
1	1.31	0.82–2.11	0.78	0.65–0.93
2	0.98	0.65–1.50	0.82	0.70–0.97
Pain/Discomfort: No				
Yes	1.66	1.13–2.44	1.17	1.02–1.33
Anxiety/Depression: No				
Yes	**2.47**	**1.74–3.49**	**1.34**	**1.18–1.52**
Characteristics	Perceived increases in body weight following a cancer diagnosis (no changes as reference)		Perceived decreases in body weight following a cancer diagnosis (no changes as reference)	
	**OR**	**CI**	**OR**	**CI**
Sociodemographic (bold as reference)				
Age	**0.96**	**0.95–0.97**	1.00	0.99–1.01
Sex: Female				
Male	**1.30**	**1.11–1.51**	0.82	0.69–0.97
Ethnicity: Any other ethnicity				
White	1.15	0.92–1.44	0.81	0.64–1.03
Highest level of education: Degree				
None	**1.31**	**1.09–1.58**	1.02	0.83–1.26
GCSE/vocational	1.08	0.90–1.30	0.89	0.73–1.08
A level	1.28	1.01–1.61	0.97	0.74–1.26
Marital status: Married/cohabiting				
Unmarried	1.03	0.89–1.19	1.06	0.91–1.25
Employment: Employed				
Unemployed	1.01	0.85–1.19	1.07	0.88–1.31
Clinical (bold as reference)				
Cancer spread: No				
Yes	**1.49**	**1.19–1.85**	**2.07**	**1.65–2.60**
Number of comorbid conditions: 3 or more				
0	**0.73**	**0.59–0.91**	**0.45**	**0.35–0.56**
1	0.76	0.62–0.93	**0.56**	**0.45–0.69**
2	0.84	0.67–1.05	0.75	0.60–0.94
Number of treatments: 3 or more				
0	**0.25**	**0.16–0.37**	0.50	0.34–0.74
1	**0.50**	**0.41–0.61**	0.75	0.61–0.93
2	**0.76**	**0.64–0.90**	0.74	0.61–0.89
Pain/Discomfort: No				
Yes	**1.44**	**1.25–1.67**	1.25	1.07–1.46
Anxiety/Depression: No				
Yes	**1.36**	**1.19–1.56**	1.06	0.91–1.24

*OR* odds ratios, *CI* confidence interval. Bold indicates significance at p<0.05 level

## Discussion

The present study used a large sample (*N* = 5835) to retrospectively assess perceived changes in health behaviours following a cancer diagnosis. Almost half of the participants in this study did not perceive that their physical activity, alcohol intake and/or body weight had changed, and more than 70% did not perceive that their diet had changed. The remaining participants in this study perceived both positive and negative changes which suggests individual differences in how a cancer diagnosis is perceived to affect these behaviours as opposed to all participants perceiving changes in the same direction.

These findings contrast with studies measuring actual changes in behaviours. For example, three longitudinal studies with sample sizes between 433 and 497 found no changes in participants’ body weight, alcohol intake and diet following a cancer diagnosis [8–10]. This difference may reflect inaccurate perceptions or may reflect that changes perceived by individuals may be subtle and not necessarily captured by standard measures of these behaviours at fixed time points. If changes are occurring in different directions for different individuals, this may also be masked by studies that simply report changes in means over time.

However, longitudinal studies have reported small changes in behaviour post-diagnosis. A secondary analysis of data from the English Longitudinal Study of Ageing reported slight increases in sedentarism (5.1 to 8.6%) and decreases in physical activity (13.2 to 9.4%) two years after a cancer diagnosis [8]. Our study found that approximately 35.9% of participants perceived decreases in their physical activity levels. Possible reasons for a reduction in physical activity may relate to physical symptoms from the disease and medical treatment side-effects [30]. The small number of individuals who reported improvements in PA may reflect individuals who received advice and support on physical activity and felt able to act on this [31, 32].

In contrast, for some individuals a cancer diagnosis may have been a teachable moment [14] which led to perceived positive changes in health behaviours, mainly healthier diets (24.3%), and reduced alcohol intake (31.3%). A cross-sectional study in the United States found that 40.4% of breast, prostate and colorectal patients reported healthier diets following a cancer diagnosis, but the study had a smaller sample size (N = 356) and did not ask about unhealthy health behaviour change, which may have led to bias [16]. Perceived improvements in eating may relate to increases in knowledge about healthy eating following diagnosis and may also indicate a general interest in this area [23]. However, it may also suggest further advice to improve may not be seen as relevant even if perceptions are inaccurate or individuals are still not consuming a diet that adheres to guidelines despite any improvements made. Since this study assessed perceived diet through several components, such as perceived red meat consumption and sugary drink intake, future research could explore perceived changes on specific dietary components.

Participants also perceived increases (27.0%) and decreases in body weight (19.2%) in this study, both of which may be associated with worse health outcomes. Reduced appetite and other side effects of treatment following a cancer diagnosis may lead to weight loss [33, 34], whereas some treatments may instead cause weight gain [35]. Weight loss could have also been intentional and positive for health outcomes, but this hypothesis was not assessed in this study. Future research should explore the differences between perceived intentional and unintentional weight loss among individuals LWBC, as well as associations with perceived weight status.

Perceptions of health behaviour change among individuals LWBC may be influenced by heterogenous factors [36]. The social determinants of health framework proposes that non-medical factors have a direct effect on individuals’ overall health, and this follows a social gradient, where individuals with lower income and from ethnic minorities face poorer health outcomes [37–39]. In this study, individuals with no education and who were unmarried, were less likely to perceive changes in their health behaviours and body weight, or perceived negative changes such as increased body weight, decreased physical activity and unhealthier diet. Not being married is typically related to having a lower quality of life in cancer patients [40] and a lack of education is related to lower health literacy [31]. Individuals in these groups may benefit from targeted support to prevent negative behavioural changes and encourage more positive changes post-diagnosis.

Younger individuals were more likely to perceive increases in physical activity and improvements in diet. Worsening health behaviours are common with ageing, and these may predict poorer physical and cognitive function, as well as increase mortality risk [41]. However, younger participants were more likely to perceive increases in body weight, which may suggest perceived improvements in physical activity and diet were inaccurate or small. Perceived increases in alcohol intake were associated with weight gain and were also more common among younger participants. Males and individuals with no education were more likely to perceive decreases in alcohol intake. A cross-sectional study of 15,199 cancer survivors found men were more likely to report being alcohol drinkers but found no association between alcohol intake and level of education [42]. Our results suggest men may perceive that they have reduced their alcohol consumption following their cancer diagnosis, but further studies are needed to confirm this and to explore potential reasons for these perceptions.

Regarding clinical characteristics, individuals with anxiety/depression and pain/discomfort were more likely to perceive changes in physical activity, diet, and body weight. A cross-sectional study found that pain/discomfort were barriers for being physically active [18]. Thus, clinicians must pay attention to these clinical characteristics since these individuals may experience heightened emotional sequelae (i.e., anxiety/depression) and pain/fatigue [43, 44], which may impact their quality of life and adherence to positive health behaviours [45]. These individuals may benefit from psychosocial interventions to help them better manage their emotional and physical symptoms [46]. For those with worse health who reported improvements in their behaviours, this may reflect an effort to manage their health through for example engaging in more physical activity. The psychosocial effects of cancer can be complex and impact people in various ways [47], and this may explain why some clinical characteristics influenced in both positive and negative ways [36].

Strengths of this study include the large sample size, use of multiple imputation, and the novel measurement of perceived changes in health behaviours following a cancer diagnosis. Limitations of this study include the representativeness of the sample since 90% of the sample were of white ethnicity and bias from using a self-report survey. For example, individuals who agree to participate in questionnaires about their health behaviours may already have an interest in improving their own health when compared to those who do not participate [48]. Additionally, the cross-sectional design of this study limits the possibility of establishing cause-effect relationships [49]. Future research could include a more representative sample, assess perceived changes at different time points and compare with actual change to better understand the accuracy of these perceptions. Cancer type was not included as a covariate in these analyses. Future studies might consider exploring if there are differences in perceptions of change based on the type of cancer diagnosed, particularly given the evidence for the role of these behaviours in cancer survivorship is stronger for certain cancers [50].

While our study focused on perceived changes post-diagnosis, other studies have explored whether individuals perceive they need to change their behaviours post-diagnosis. One study in the same population identified that most individuals do not perceive they need to change their diet [23], whereas another study found most people living with overweight/obesity perceive they do need to lose weight [48]. Understanding both how an individual perceives they have changed, as well as how they perceive they need to change going forward, is likely to be important for interventions promoting health behaviours.

## Conclusions

Approximately half of participants did not perceive changes in their physical activity, body weight and alcohol intake, and most did not perceive changes in their diet following a diagnosis of cancer. Individuals with no education and who were unmarried, with anxiety/depression, and pain/discomfort, were more likely to perceive changes, but these were not in the same direction for all groups and behaviours. These characteristics could help healthcare professionals to target support at those most at risk of perceiving negative changes in the behaviour of interest. Asking participants about their perceptions could also inform effective health promotion conversations. Future research in diverse samples should also explore how perceptions impact actual behaviour and outcomes, as well as reasons behind perceived changes in more diverse samples, to help us better understand how to target and design health behaviour change interventions for individuals living with and beyond cancer.

## Supplementary Information

Below is the link to the electronic supplementary material.

ESM 1 (DOCX 30.6 KB)

## Data Availability

The datasets generated during and/or analysed during the current study are available from the corresponding author on reasonable request .

## References

[1] Sung H, Ferlay J, Siegel RL, Laversanne M, Soerjomataram I, Jemal A, Bray F (2021) Global cancer statistics 2020: GLOBOCAN estimates of incidence and mortality worldwide for 36 cancers in 185 countries. CA Cancer J Clin 71(3):209–249. 10.3322/caac.21660 33538338 10.3322/caac.21660

[2] Smittenaar CR, Petersen KA, Stewart K, Moitt N (2016) Cancer incidence and mortality projections in the UK until 2035. Br J Cancer 115(9):1147–1155. 10.1038/bjc.2016.304 27727232 10.1038/bjc.2016.304 PMC5117795

[3] Cancer Research UK (2021) Cancer survival statistics. Retrieved July 20, 2022, from https://www.cancerresearchuk.org/health-professional/cancer-statistics/survival#heading-Zero

[4] American Cancer Society (2022) Long-term side effects of cancer. Retrieved July 20, 2022, from https://www.cancer.org/treatment/survivorship-during-and-after-treatment/long-term-health-concerns/long-term-side-effects-of-cancer.html

[5] World Cancer Research Fund (2018) Diet, nutrition, physical activity, and cancer: a global perspective. Continuous Update Project Expert Report 2018

[6] Park B, Kong SY, Kim J, Kim Y, Park IH, Jung SY, Lee ES (2015) Health behaviors of cancer survivors in nationwide cross-sectional survey in Korea: higher alcohol drinking, lower smoking, and physical inactivity pattern in survivors with higher household income. Medicine (Baltimore) 94(31):e1214. 10.1097/md.0000000000001214 26252280 10.1097/MD.0000000000001214 PMC4616611

[7] World Cancer Research Fund. (n.d.). Cancer survivors. Retrieved April, 2022, from https://www.wcrf.org/diet-activity-and-cancer/global-cancer-update-programme/cancer-survivors/

[8] Williams K, Steptoe A, Wardle J (2013) Is a cancer diagnosis a trigger for health behaviour change? Findings from a prospective, population-based study. Br J Cancer 108(11):2407–2412. 10.1038/bjc.2013.254 23695026 10.1038/bjc.2013.254 PMC3681023

[9] Bidstrup PE, Dalton SO, Christensen J, Tjonneland A, Larsen SB, Karlsen R, ... Johansen C (2013) Changes in body mass index and alcohol and tobacco consumption among breast cancer survivors and cancer-free women: a prospective study in the Danish diet, cancer and health cohort. Acta Oncol 52(2):327–335. 10.3109/0284186x.2012.746466 10.3109/0284186X.2012.746466 23244678

[10] Newson JT, Huguet N, Ramage-Morin PL, McCarthy MJ, Bernier J, Kaplan MS, McFarland BH (2012) Health behaviour changes after diagnosis of chronic illness among Canadians aged 50 or older. Health Rep 23(4):49–53 23356045 PMC4427234

[11] Tollosa DN, Holliday E, Hure A, Tavener M, James EL (2020) A 15-year follow-up study on long-term adherence to health behaviour recommendations in women diagnosed with breast cancer. Breast Cancer Res Treat 182(3):727–738. 10.1007/s10549-020-05704-4 32535764 10.1007/s10549-020-05704-4

[12] Barbosa A, Costa AR, Fontes F, Dias T, Pereira S, Lunet N (2019) Changes in health behaviours and body mass index after a breast cancer diagnosis: results from a prospective cohort study. Eur J Cancer Prev 28(4):330–337. 10.1097/cej.0000000000000469 30272598 10.1097/CEJ.0000000000000469

[13] Bluethmann SM, Bartholomew LK, Murphy CC, Vernon SW (2017) Use of theory in behavior change interventions. Health Educ Behav 44(2):245–253. 10.1177/1090198116647712 10.1177/1090198116647712 PMC5503486 27226430

[14] McBride CM, Ostroff JS (2003) Teachable moments for promoting smoking cessation: the context of cancer care and survivorship. Cancer Control 10(4):325–333. 10.1177/107327480301000407 12915811 10.1177/107327480301000407

[15] Tollosa DN, Holliday E, Hure A, Tavener M, James EL (2020) Multiple health behaviors before and after a cancer diagnosis among women: a repeated cross-sectional analysis over 15 years. Cancer Med 9(9):3224–3233. 10.1002/cam4.2924 32134568 10.1002/cam4.2924 PMC7196049

[16] Patterson RE, Neuhouser ML, Hedderson MM, Schwartz SM, Standish LJ, Bowen DJ (2003) Changes in diet, physical activity, and supplement use among adults diagnosed with cancer. J Am Diet Assoc 103(3):323–328. 10.1053/jada.2003.50045 12616253 10.1053/jada.2003.50045

[17] Beeken RJ, Williams K, Wardle J, Croker H (2016) “What about diet?” A qualitative study of cancer survivors’ views on diet and cancer and their sources of information. Eur J Cancer Care (Engl) 25(5):774–783. 10.1111/ecc.12529 27349812 10.1111/ecc.12529 PMC4995727

[18] Keaver L, McGough AM, Du M, Chang W, Chomitz V, Allen JD, Zhang FF (2021) Self-reported changes and perceived barriers to healthy eating and physical activity among global breast cancer survivors: results from an exploratory online novel survey. Journal of the Academy of Nutrition and Dietetics 121(2):233-241.e238. 10.1016/j.jand.2020.09.031 33109503 10.1016/j.jand.2020.09.031 PMC11217928

[19] Conner M, Norman P (2022) Understanding the intention-behavior gap: the role of intention strength. Front Psychol 13:923464. 10.3389/fpsyg.2022.923464 35992469 10.3389/fpsyg.2022.923464 PMC9386038

[20] Stevens C, Vrinten C, Smith SG, Waller J, Beeken RJ (2018) Determinants of willingness to receive healthy lifestyle advice in the context of cancer screening. Br J Cancer 119(2):251–257. 10.1038/s41416-018-0160-4 29991698 10.1038/s41416-018-0160-4 PMC6048170

[21] Kiviniemi MT, Rothman AJ (2008) What do people think about changes in health behaviors? Differential perceptions of consequences of increases and decreases in health behaviors. Psychol Health 23(7):867–885. 10.1080/14768320701360500 19802395 10.1080/14768320701360500 PMC2753289

[22] Warmoth K, Tarrant M, Abraham C, Lang IA (2016) Older adults’ perceptions of ageing and their health and functioning: a systematic review of observational studies. Psychol Health Med 21(5):531–550. 10.1080/13548506.2015.1096946 26527056 10.1080/13548506.2015.1096946

[23] Smith S, Fisher A, Lally PJ, Croker HA, Roberts A, Conway RE, Beeken RJ (2024) Perceiving a need for dietary change in adults living with and beyond cancer: a cross-sectional study. Cancer Med 13(4):e7073. 10.1002/cam4.7073 38457197 10.1002/cam4.7073 PMC10922024

[24] Beeken RJ, Croker H, Heinrich M, Smith L, Williams K, Hackshaw A, Fisher A (2016) Study protocol for a randomised controlled trial of brief, habit-based, lifestyle advice for cancer survivors: exploring behavioural outcomes for the advancing Survivorship Cancer Outcomes Trial (ASCOT). BMJ open 6(11):e011646. 10.1136/bmjopen-2016-011646 27881518 10.1136/bmjopen-2016-011646 PMC5178807

[25] Feng YS, Kohlmann T, Janssen MF, Buchholz I (2021) Psychometric properties of the EQ-5D-5L: a systematic review of the literature. Qual Life Res 30(3):647–673. 10.1007/s11136-020-02688-y 33284428 10.1007/s11136-020-02688-y PMC7952346

[26] IBM Corp (2016) IBM SPSS Statistics for Macintosh, Version 25.0. IBM Corp, Armonk

[27] Dong Y, Peng CY (2013) Principled missing data methods for researchers. Springerplus 2(1):222. 10.1186/2193-1801-2-222 23853744 10.1186/2193-1801-2-222 PMC3701793

[28] Sterne JA, White IR, Carlin JB, Spratt M, Royston P, Kenward MG, Carpenter JR (2009) Multiple imputation for missing data in epidemiological and clinical research: potential and pitfalls. BMJ 338:b2393. 10.1136/bmj.b2393 19564179 10.1136/bmj.b2393 PMC2714692

[29] Kim JH (2019) Multicollinearity and misleading statistical results. Korean J Anesthesiol 72(6):558–569. 10.4097/kja.19087 31304696 10.4097/kja.19087 PMC6900425

[30] Bränström R, Petersson LM, Saboonchi F, Wennman-Larsen A, Alexanderson K (2015) Physical activity following a breast cancer diagnosis: Implications for self-rated health and cancer-related symptoms. Eur J Oncol Nurs 19(6):680–685. 10.1016/j.ejon.2015.04.008 26051073 10.1016/j.ejon.2015.04.008

[31] Holden CE, Wheelwright S, Harle A, Wagland R (2021) The role of health literacy in cancer care: a mixed studies systematic review. PLoS ONE 16(11):e0259815. 10.1371/journal.pone.0259815 34767562 10.1371/journal.pone.0259815 PMC8589210

[32] Buja A, Rabensteiner A, Sperotto M, Grotto G, Bertoncello C, Cocchio S, Baldo V (2020) Health literacy and physical activity: a systematic review. J Phys Act Health 17(12):1259–1274. 10.1123/jpah.2020-0161 33129198 10.1123/jpah.2020-0161

[33] Shang L, Hattori M, Fleming G, Jaskowiak N, Hedeker D, Olopade OI, Huo D (2021) Impact of post-diagnosis weight change on survival outcomes in black and white breast cancer patients. Breast Cancer Res 23(1):18. 10.1186/s13058-021-01397-9 33541403 10.1186/s13058-021-01397-9 PMC7863526

[34] Ee C, Cave AE, Naidoo D, Bilinski K, Boyages J (2020) Weight before and after a diagnosis of breast cancer or ductal carcinoma in situ: a national Australian survey. BMC Cancer 20(1):113. 10.1186/s12885-020-6566-4 32075592 10.1186/s12885-020-6566-4 PMC7031941

[35] Anderson AS, Martin RM, Renehan AG, Cade J, Copson ER, Cross AJ, Saxton JM (2021) Cancer survivorship, excess body fatness and weight-loss intervention-where are we in 2020? British journal of cancer 124(6):1057–1065. 10.1038/s41416-020-01155-2 33235316 10.1038/s41416-020-01155-2 PMC7961062

[36] Hoedjes M, Nijman I, Hinnen C (2022) Psychosocial determinants of lifestyle change after a cancer diagnosis: a systematic review of the literature. Cancers 14(8):2026. 10.3390/cancers14082026 35454932 10.3390/cancers14082026 PMC9032592

[37] Iacobucci G (2013) Most British non-white groups are less healthy than white people, census data show. BMJ : British Medical Journal 347:f6160. 10.1136/bmj.f6160 24124174 10.1136/bmj.f6160

[38] Marmot M, Bell R (2012) Fair society, healthy lives. Public Health 126(Suppl 1):S4-s10. 10.1016/j.puhe.2012.05.014 22784581 10.1016/j.puhe.2012.05.014

[39] World Health Organization (n.d.) Social determinants of health. Retrieved July 20, 2022, from https://www.who.int/health-topics/social-determinants-of-health#tab=tab_1

[40] Corovic S, Vucic V, Mihaljevic O, Djordjevic J, Colovic S, Radovanovic S, Milovanovic O (2023) Social support score in patients with malignant diseases—with sociodemographic and medical characteristics. Frontiers in Psychology 14:1160020. 10.3389/fpsyg.2023.1160020 37325739 10.3389/fpsyg.2023.1160020 PMC10267316

[41] Khaw KT, Wareham N, Bingham S, Welch A, Luben R, Day N (2008) Combined impact of health behaviours and mortality in men and women: the EPIC-Norfolk prospective population study. PLoS Med 5(1):e12. 10.1371/journal.pmed.0050012 18184033 10.1371/journal.pmed.0050012 PMC2174962

[42] Shi M, Luo C, Oduyale OK, Zong X, LoConte NK, Cao Y (2023) Alcohol consumption among adults with a cancer diagnosis in the all of us research program. JAMA Netw Open 6(8):e2328328. 10.1001/jamanetworkopen.2023.28328 37561459 10.1001/jamanetworkopen.2023.28328 PMC10415957

[43] Karunanithi G, Sagar RP, Joy A, Vedasoundaram P (2018) Assessment of psychological distress and its effect on quality of life and social functioning in cancer patients. Indian J Palliat Care 24(1):72–77. 10.4103/ijpc.Ijpc_104_17 29440811 10.4103/IJPC.IJPC_104_17 PMC5801634

[44] Narayanan V, Koshy C (2009) Fatigue in cancer: a review of literature. Indian J Palliat Care 15(1):19–25. 10.4103/0973-1075.53507 20606851 10.4103/0973-1075.53507 PMC2886215

[45] Kennedy F, Lally P, Miller NE, Conway RE, Roberts A, Croker H, Beeken RJ (2023) Fatigue, quality of life and associations with adherence to the world cancer research fund guidelines for health behaviours in 5835 adults living with and beyond breast, prostate and colorectal cancer in England: a cross-sectional study. Cancer medicine 12(11):12705–12716. 10.1002/cam4.5899 37021752 10.1002/cam4.5899 PMC10278485

[46] Tack L, Schofield P, Boterberg T, Chandler R, Parris CN, Debruyne PR (2022) Psychosocial care after cancer diagnosis: recent advances and challenges. Cancers 14(23):5882. 10.3390/cancers14235882 36497363 10.3390/cancers14235882 PMC9739074

[47] Park CL, Gaffey AE (2007) Relationships between psychosocial factors and health behavior change in cancer survivors: an integrative review. Ann Behav Med 34(2):115–134. 10.1007/bf02872667 17927551 10.1007/BF02872667

[48] Heuchan GN, Lally PJ, Beeken RJ, Fisher A, Conway RE (2023) Perception of a need to change weight in individuals living with and beyond breast, prostate and colorectal cancer: a cross-sectional survey. J Cancer Surviv. 10.1007/s11764-023-01333-0 36701100 10.1007/s11764-023-01333-0 PMC11081928

[49] Wang X, Cheng Z (2020) Cross-sectional studies: strengths, weaknesses, and recommendations. Chest 158(1, Supplement):S65–S71. 10.1016/j.chest.2020.03.012 32658654 10.1016/j.chest.2020.03.012

[50] Clinton SK, Giovannucci EL, Hursting SD (2020) The world cancer research fund/american institute for cancer research third expert report on diet, nutrition, physical activity, and cancer: impact and future directions. J Nutr 150(4):663–671. 10.1093/jn/nxz268 31758189 10.1093/jn/nxz268 PMC7317613

