# Supplementary material for: Perceived changes in health behaviours and body weight in response to a cancer diagnosis among individuals living with and beyond breast, prostate, and colorectal cancer in the UK: a cross-sectional study
Source: Support Care Cancer. 2025 Mar 4;33(3):247. doi: 10.1007/s00520-025-09305-z (PMC11880087; doi:10.1007/s00520-025-09305-z)
Supplement: Supplementary file 1 — (DOCX 30.6 KB) [file 520_2025_9305_MOESM1_ESM.docx]

**Supplementary Materials**

Supplementary Table 1: Logistic regressions in physical activity and diet for completers dataset (*N* = 3729)

| **Characteristics** | **Perceived increases in physical activity following a cancer diagnosis (no changes as reference)** | | **Perceived decreases in physical activity following a cancer diagnosis (no changes as reference)** | | | |
| --- | --- | --- | --- | --- | --- | --- |
|  | **OR** | **CI** | **OR** | | **CI** | |
| ***Sociodemographic (bold as reference)*** |  |  |  | |  | |
| **Age** | 0.96 | 0.94-0.97 | 0.99 | | 0.98-1.00 | |
| **Sex: Female** |  |  |  | |  | |
| Male | 0.81 | 0.63-1.04 | 1.27 | | 1.08-1.51 | |
| **Ethnicity: Any other ethnicity** |  |  |  | |  | |
| White | 0.76 | 0.56-1.03 | 0.88 | | 0.69-1.13 | |
| **Highest level of education: Degree** |  |  |  | |  | |
| None | 0.57 | 0.43-0.76 | 1.33 | | 1.09-1.63 | |
| GCSE/vocational | 0.59 | 0.46-0.75 | 1.10 | | 0.91-1.33 | |
| A level | 0.67 | 0.49-0.93 | 1.17 | | 0.91-1.50 | |
| **Marital status: Married/cohabiting** |  |  |  | |  | |
| Unmarried | 1.07 | 0.86-1.34 | 1.32 | | 1.13-1.54 | |
| **Employment: Employed** |  |  |  | |  | |
| Unemployed | 1.56 | 1.22-2.00 | 1.28 | | 1.07-1.55 | |
| ***Clinical (bold as reference)*** |  |  |  | |  | |
| **Cancer spread: No** |  |  |  | |  |  |
| Yes | 1.28 | 0.92-1.78 | 2.14 | | 1.71-2.68 | |
| **Number of comorbid conditions: 3 or more** |  |  |  | |  | |
| 0 | 1.03 | 0.71-1.48 | 0.57 | | 0.45-0.72 | |
| 1 | 1.00 | 0.70-1.43 | 0.59 | | 0.48-0.74 | |
| 2 | 1.04 | 0.70-1.54 | 0.70 | | 0.56-0.89 | |
| **Number of treatments: 3 or more** |  |  |  | |  | |
| 0 | 0.85 | 0.49-1.46 | 0.25 | | 0.16-0.39 | |
| 1 | 0.91 | 0.68-1.22 | 0.56 | | 0.45-0.68 | |
| 2 | 0.95 | 0.74-1.22 | 0.72 | | 0.60-0.87 | |
| **Pain/Discomfort: No** |  |  |  | |  | |
| Yes | 1.06 | 0.86-1.30 | 2.00 | | 1.72-2.33 | |
| **Anxiety/Depression: No** |  |  |  | |  | |
| Yes | 1.40 | 1.14-1.71 | 1.67 | | 1.45-1.93 | |
| **Characteristics** | **Perceived healthier diet following a cancer diagnosis (no changes as reference)** | | **Perceived unhealthier diet following a cancer diagnosis (no changes as reference)** | | | |
|  | **OR** | **CI** | **OR** | **CI** | | |
| ***Sociodemographic (bold as reference)*** |  |  |  |  | | |
| **Age** | 0.96 | 0.95-0.97 | 0.99 | 0.97-1.01 | | |
| **Sex: Female** |  |  |  |  | | |
| Male | 1.23 | 1.02-1.47 | 0.90 | 0.60-1.35 | | |
| **Ethnicity: Any other ethnicity** |  |  |  |  | | |
| White | 0.48 | 0.38-0.60 | 0.60 | 0.36-1.00 | | |
| **Highest level of education: Degree** |  |  |  |  | | |
| None | 1.06 | 0.86-1.30 | 1.98 | 1.22-3.22 | | |
| GCSE/vocational | 0.94 | 0.78-1.13 | 1.28 | 0.80-2.06 | | |
| A level | 0.76 | 0.59-0.98 | 1.43 | 0.81-2.52 | | |
| **Marital status: Married/cohabiting** |  |  |  |  | | |
| Unmarried | 1.11 | 0.94-1.30 | 2.42 | 1.72-3.38 | | |
| **Employment: Employed** |  |  |  |  | | |
| Unemployed | 1.09 | 0.90-1.31 | 0.89 | 0.58-1.36 | | |
| ***Clinical (bold as reference)*** |  |  |  |  | | |
| **Cancer spread: No** |  |  |  |  | | |
| Yes | 1.45 | 1.16-1.81 | 3.38 | 2.27-5.01 | | |
| **Number of comorbid conditions: 3 or more** |  |  |  |  | | |
| 0 | 0.74 | 0.58-0.95 | 0.71 | 0.43-1.17 | | |
| 1 | 0.75 | 0.59-0.95 | 0.52 | 0.32-0.84 | | |
| 2 | 0.82 | 0.64-1.07 | 0.70 | 0.42-1.14 | | |
| **Number of treatments: 3 or more** |  |  |  |  | |  |
| 0 | 0.58 | 0.37-0.90 | 0.33 | 0.08-1.42 | | |
| 1 | 0.79 | 0.63-0.98 | 0.91 | 0.57-1.46 | | |
| 2 | 0.92 | 0.76-1.11 | 0.85 | 0.55-1.30 | | |
| **Pain/Discomfort: No** |  |  |  |  | | |
| Yes | 0.95 | 0.81-1.11 | 2.03 | 1.34-3.10 | | |
| **Anxiety/Depression: No** |  |  |  |  | | |
| Yes | 1.19 | 1.02-1.38 | 1.78 | 1.26-2.50 | | |

Note: OR = odds ratios; CI = confidence interval.

Supplementary Table 2: Logistic regressions in alcohol intake and body weight for completers dataset (*N* = 3729)

| **Characteristics** | **Perceived increases in alcohol intake following a cancer diagnosis (no changes as reference)** | | **Perceived decreases in alcohol intake following a cancer diagnosis (no changes as reference)** | |
| --- | --- | --- | --- | --- |
|  | **OR** | **CI** | **OR** | **CI** |
| ***Sociodemographic (bold as reference)*** |  |  |  |  |
| **Age** | 0.96 | 0.94-0.98 | 0.98 | 0.97-0.99 |
| **Sex: Female** |  |  |  |  |
| Male | 0.93 | 0.57-1.52 | 1.45 | 1.22-1.73 |
| **Ethnicity: Any other ethnicity** |  |  |  |  |
| White | 1.35 | 0.60-3.01 | 0.52 | 0.40-0.67 |
| **Highest level of education: Degree** |  |  |  |  |
| None | 0.84 | 0.46-1.53 | 1.63 | 1.34-1.99 |
| GCSE/vocational | 1.03 | 0.67-1.57 | 1.00 | 0.83-1.19 |
| A level | 1.00 | 0.56-1.80 | 1.11 | 0.87-1.41 |
| **Marital status: Married/cohabiting** |  |  |  |  |
| Unmarried | 1.28 | 0.86-1.90 | 0.99 | 0.84-1.15 |
| **Employment: Employed** |  |  |  |  |
| Unemployed | 1.44 | 0.92-2.25 | 1.11 | 0.93-1.33 |
| ***Clinical (bold as reference)*** |  |  |  |  |
| **Cancer spread: No** |  |  |  |  |
| Yes | 1.30 | 0.74-2.29 | 1.75 | 1.41-2.19 |
| **Number of comorbid conditions: 3 or more** |  |  |  |  |
| 0 | 2.39 | 1.08-5.30 | 0.85 | 0.67-1.08 |
| 1 | 2.36 | 1.08-5.12 | 0.91 | 0.73-1.14 |
| 2 | 1.84 | 0.79-4.28 | 0.94 | 0.73-1.20 |
| **Number of treatments: 3 or more** |  |  |  |  |
| 0 | 0.22 | 0.03-1.68 | 0.42 | 0.29-0.64 |
| 1 | 1.22 | 0.72-2.08 | 0.75 | 0.61-0.92 |
| 2 | 0.97 | 0.61-1.55 | 0.89 | 0.74-1.07 |
| **Pain/Discomfort: No** |  |  |  |  |
| Yes | 1.44 | 0.95-2.17 | 1.16 | 1.00-1.35 |
| **Anxiety/Depression: No** |  |  |  |  |
| Yes | 2.27 | 1.53-3.35 | 1.41 | 1.22-1.63 |
| **Characteristics** | **Perceived increases in body weight following a cancer diagnosis (no perceived changes as reference)** | | **Perceived decreases in body weight following a cancer diagnosis (no perceived changes as reference)** | |
|  | **OR** | **CI** | **OR** | **CI** |
| ***Sociodemographic (bold as reference)*** |  |  |  |  |
| **Age** | 0.96 | 0.95-0.96 | 0.99 | 0.98-1.00 |
| **Sex: Female** |  |  |  |  |
| Male | 1.34 | 1.11-1.61 | 0.84 | 0.68-1.02 |
| **Ethnicity: Any other ethnicity** |  |  |  |  |
| White | 1.11 | 0.86-1.43 | 0.82 | 0.62-1.08 |
| **Highest level of education: Degree** |  |  |  |  |
| None | 1.37 | 1.10-1.69 | 1.00 | 0.79-1.26 |
| GCSE/vocational | 1.06 | 0.88-1.29 | 0.87 | 0.70-1.09 |
| A level | 1.26 | 0.98-1.61 | 0.87 | 0.65-1.18 |
| **Marital status: Married/cohabiting** |  |  |  |  |
| Unmarried | 1.00 | 0.84-1.18 | 1.11 | 0.93-1.34 |
| **Employment: Employed** |  |  |  |  |
| Unemployed | 1.10 | 0.91-1.33 | 1.12 | 0.89-1.40 |
| ***Clinical (bold as reference)*** |  |  |  |  |
| **Cancer spread: No** |  |  |  |  |
| Yes | 1.44 | 1.13-1.83 | 2.00 | 1.54-2.59 |
| **Number of comorbid conditions: 3 or more** |  |  |  |  |
| 0 | 0.72 | 0.55-0.93 | 0.40 | 0.30-0.53 |
| 1 | 0.76 | 0.60-0.97 | 0.52 | 0.40-0.66 |
| 2 | 0.87 | 0.67-1.14 | 0.72 | 0.55-0.94 |
| **Number of treatments: 3 or more** |  |  |  |  |
| 0 | 0.26 | 0.16-0.42 | 0.53 | 0.33-0.86 |
| 1 | 0.52 | 0.42-0.65 | 0.75 | 0.59-0.96 |
| 2 | 0.73 | 0.60-0.88 | 0.80 | 0.64-1.00 |
| **Pain/Discomfort: No** |  |  |  |  |
| Yes | 1.45 | 1.24-1.71 | 1.21 | 1.01-1.45 |
| **Anxiety/Depression: No** |  |  |  |  |
| Yes | 1.33 | 1.14-1.55 | 1.11 | 0.94-1.33 |

Note: OR = odds ratios; CI = confidence interval.
